# The American Association for the Cure of Inebriates

**Published:** 1879-07

**Authors:** 


### THE AMERICAN ASSOCIATION FOR THE CURE OF INEBRIATES.

New York, May 13th.

*First Day.*—The Association met in the parlors of the Young Men's Christian Association, and was called to order by the President, Dr. Willard Parker, of New York. In his opening address the President referred to the magnitude of the study of inebriety, and the increasing interest apparent among medical men all over the country. The confusion of both theory and prevalent opinion naturally followed the first study of all great topics, and was really a hopeful sign. The past year had brought the most convincing proofs that the inebriate asylum movement was destined to meet the question of inebriety in a more practical manner than any other. Our duty was clear: as long as the laws endorsed the free use of alcohol, so long would there be a necessity for asylums, both inebriate and insane. When the public recognized the necessity of inebriate hospitals, then the need of palace insane asylums would be lessened. He believed he was safe in saying that fully one-third of all our insane might have been saved, had they been placed in inebriate hospitals early in their history. The inebriety from which they suffered would have been checked before it went on to insanity. The Association was destined to occupy a very wide place in educating the public and developing the laws which controlled the complex disorder. In concluding, Dr. Parker urged that the Association should not spend time in discussing any of the theories urged in opposition to its work.

After the preliminary business, Dr. J. B. Mattison read a very excellent paper on "Chloral Inebriety."

In the discussion which followed, Dr. Parrish related a case of a hypochondriac who used opium and chloral alternately, and who made many ineffectual attempts to stop. The withdrawal of these drugs beyond a certain point was attended with severe prostration and violent fever. The doctor had also made the effort, and concluded it was great wisdom to begin again. He mentioned this case as anomalous, and as indicating a condition in which the drug seemed to be demanded for life.

Dr. Parker had noticed a similar case.

Dr. Mason had seen a case where morphia was used for years, and the slightest increase or diminution would provoke violent symptoms which necessitated a return to the drug. The patient was living and in fair health.

Dr. Mattison suggested that where opium was used in conjunction with the bromides, the danger of continuing its use was greatly lessened, and in cutting down the use of opium the bromides were most serviceable.

Dr. G. M. Beard, of New York, had had a large experience in the use of narcortics, and had found that they were very often antidotes one to the other. In very troublesome cases of insomnia, he used always combinations of opium, bromides, alcohol, and other narcotics, and by alternating them he had often accomplished his purpose without any entailment or danger. He thought chloral as a medicine would seldom be followed by chloral inebriety if used in that way. In all cases where chloral was used, it should be watched very carefully.

LOSS OF MEMORY AND CONSCIOUSNESS IN INEBRIETY.

Dr T. D. Crothers of Hartford, then read a paper upon the above subject, which was a study of six cases of inebriety, in which the patients had total blanks of memory and consciousness while drinking, and yet went about and transacted business, and gave no evidence of that physical condition. In one of the cases mentioned, the patient, while in that condition, witnessed an assault and murder, and testified clearly the next day before the coroner's jury, and on the third day recovered his mind and had no recollection of it whatever. He was arrested, and was supposed to have assumed that state for the purpose of shielding the prisoner.

Another case was mentioned, of a business man who lost all memory of events while working over his books. Two days after he awoke in a distant city, and all the interval was a blank, although he had made several important business transactions and seemed perfectly conscious of all his circumstances.

The doctor indicated that cerebral automatism was present in all of these cases, and concluded with a mention of some other cases and some of the medico-legal bearings of these cases.

In the discussion which followed, Dr. G. M. Beard said the cases reported by Dr. Crothers were clearly those of cerebral trance, a suspension of some faculties and intensification of others. That was one phase of involuntary life which was rarely studied by physiologists, and was always full of mystery.

Dr. Willet mentioned a case of a man who, while drinking, supposed that he was married, and related all the circumstances with great minuteness, offering to go on the stand and make oath to them. The history showed that it was all delusion; and from that he argued at some length of the danger of accepting the testimony of inebriates, unless verified by other circumstances, in important trials. He would not infer that they wilfully falsified, but the liability to delusion was great.

Dr. Mason was confident that the testimony of drinking men was always more or less unreliable. He referred to a case of a man of excellent character for veracity, but who had made positive statements, and denied them equally as positively the next day. He was drinking when he made the first statement, although apparently conscious of the import and meaning of his words; the next day he was clearer, and had no reccollection of his words the day before. The doctor mentioned a second case of a man who was constantly under some delusion when drinking, and yet it was not observed by his friends, and he gave no evidence of any mental disturbance.

Dr. Parrish thought that such cases were examples of paralysis of memory and will. He had seen many similar cases among the insane and idiotic. The function of memory was suddenly cut off, and a fair degree of intelligence left, and yet give no evidence of his real condition. Such cases were full of interest, and were of the greatest practical importance medico-legally.

Dr. Elisha Harris said Dr. Crothers had opened up a new field in his discussion of these cases, and that it threw a flood of light on many of the perplexing problems of the day. They were all types of many criminal cases, where morbid impulses had resulted in morbid cerebration, often traced to inheritance or some exciting cause. The register of memory was so impaired as to be irresponsible. He had seen some remarkable cases where the memory was a total blank. One, of a man who, while drinking, committed a murder, and, when arrested, he recovered his senses, but never could recall the events of the murder in any way. The doctor had seen other cases in which he firmly believd the person had no recollection of any of the events, and had they been well understood, they would not have been consigned to prison. He would not excuse any one for crime which they might have prevented, but these cases should be studied more carefully, and then we should understand the measure of responsibility, and do more exact justice to both the criminal and the outraged rights of society.

Medico-legally, we could not estimate their importance. Inebriety ramified in every neighborhood in the land, and its effects were felt by every section; yet the public were more or less indifferent, and failed to recognize the great fact that those cases should be studied in hospitals, and treated as diseased men. As a measure of economy alone, it would be a great advantage. These cases indicated how wide a field yet remained to be studied, and how many problems of both criminality and inebriety would be solved when we understood them.

The public were awakening to the importance of comprehending inebriety and its practical management.

The Association then adjourned.

WEDNESDAY, May 14th.

*Second Day.*—The Association was called to order by the President.

The first order of business was reports from the Secretary, Dr. T. D. Crothers, of Hartford, Conn., of which the following is an abstract:

The discussions of the effect of alcohol on society to day were marked by a vague uncertainty and a changing restlessness, unnoticed before. The increased publication of books, papers and sermons, advocating many different theories and opinions, together with the temperance revivals which had sprung up in all parts of the country, enlisting the press, and rousing up the church, followed by organized societies pledged to carry on the work (all having one common purpose—the suppression of the evils following the use of alcohol), were the most significant signs of the times, and indicated clearly a great upheaval of opinion, to be followed by a wider comprehension of those evils and their remedies.

The establishing of inebriate asylums in the midst of opposition and credulity had gone on quietly in the wake of that continuous agitation, gathering friends and influence wherever the subject and its wants are realized.

The narrow prejudice and ignorant opposition had only served to bring out more prominently the principles upon which they were founded, and behind all the clamor and sneer there was an under-current of facts (increasing every year), pointing distinctly to those asylums for a solution of the many problems of inebriety. Of over thirty inebriate asylums established in this country during the past quarter of a century, only four had suspended and gone out of existence. Considering that they were all experimental, and working without experience or precedent, and without the sympathy and co-operation of the public, their success might safely challenge comparison with any other charity of the age.

It is a well-recognized fact that the asylum treatment of inebriety was more difficult than that of insanity, and had those asylums not met a necessity as imperative as quarantine statine stations for infectious diseases, or hospitals for the insane, they would have all failed long ago. The early history of insane asylums was marked by many failures and imperfections, but the principles did not change. The conceptions of the work and the application of its principles might be wanting, but the necessity and value was the same. The necessity of hospital treatment for inebriety was established beyond all question. Within two years a very significant movement had begun, which was the commencement of a great revolution of public opinion in regard to asylums.

There had been opened in this country, within this time, over a thousand temporary lodging-houses and eating rooms for inebriates—places where the poor, homeless victim, after he had signed the pledge, could be taken and cared for until he was able to go out sober and help himself.

Some of those places had five or six beds, others less. Most of them were free. Some charged a few cents, and trusted the inebriate to pay. Many of them were connected with temperance coffee-rooms, and were scarcely known. Some of the temperance eating-rooms had the names of benevolent people, who would give a room and bed to any poor worthy inebriate who was making an effort to get well. In those places they recognized the value of physical aid, and the necessity of food and rest, before the diseased will could be restored. The pledge was first given, then the physical wants were supplied. The comforts of home and food were furnished either free, or at a cost that was merely nominal, and often clothing was also furnished. Conversation, prayer, advice, personal influence of some friend, watching and protection from all associations, and other temporary means, were employed. Many of those places were managed by societies and reformed inebriates, others by women or churches. The Women's Temperance Union and the reformed clubs seemed to sustain the most of those places. In some cities single individuals were supporting little homes of this character, and the purpose of all—to shield and protect the inebriate—was one of the fundamental principles upon which inebriate asylums were based. Without any special notice, and almost unknown in the cities and towns where they existed, those initial asylums were rapidly forming public sentiment, and preparing for a larger and more enduring work in asylums properly organized.

The value of one day's restraint in those homes would bring the most positive proof of the greater good coming from a longer time, with more perfect care and attention. If good food and quiet rest would help to overcome the diseased impulse, it was only a step to realize the value of months of such surroundings, and the possibility of permanent recovery. Those homes were rapidly increasing, and following the track of the great revivals, and they were literally the first efforts of the masses to treat inebriety by rational means. From every one would go out an influence that would far transcend the individual good they could accomplish. The public were ripe for some practical methods of reaching this disorder. A small number of asylums were at work like the videttes of an advancing army. Practical men, both in and out of those asylums, recognized the possibility of making all this vast tide of inebriety support itself in hospitals sustained by law and public sympathy. All the indications were unmistakable, that behind the noise and confusion would be seen the reign of law and growth of homes and hospitals that should meet the demands of the inebriate. The medical profession had also agitated the subject, and during the past year over twenty different papers had been written and read on alcohol and its effects on society, before medical societies in this country, and from all sides came the most cheering proofs that the work of the association was scarcely begun. . . . .

Dr. Elisha Chenery, of Boston, then read a paper on the effect of alcohol on offspring, in which he showed the effect of alcohol on the blood-corpuscles, with the changes of tissue, and the pathological conditions which followed. It was a very clear presentation of all the latest facts on the action of alcohol, and the heredity of alcoholized condition.

The paper was very ably discussed by Drs. Willard Parker, Parrish, Mattison, Willett, Mason, and others.

Dr. George M. Beard read a very able paper on some forms of neurasthenia resulting in inebriety. Rev. John Willett followed with a paper on alcohol and its origin and character, as both a beverage and medicine. These papers were discussed at some length; after which several papers were read by title and referred to appropriate committees.

